# New Spectrophotometric Estimation of Ornidazole Tablets Employing Urea as a Hydrotropic Solubilizing Additive

**DOI:** 10.4103/0250-474X.65024

**Published:** 2010

**Authors:** R. K. Maheshwari, V. K. Srivastav, R. P. Prajapat, Anshu Jain, P. Kamaria, S. Sahu

**Affiliations:** Department of Pharmacy, Shri Govindram Seksaria Institute of Technology and Sciences; 23, Park Road, Indore-452003, India

**Keywords:** Hydrotropy, ornidazole, urea, spectrophotometric analysis

## Abstract

Quantitative spectrophotometric analysis of poorly water-soluble drugs involves use of various organic solvents. Major drawbacks of organic solvents include high cost, volatility and toxicity. Safety of analyzer is affected by toxicity of the solvent used. In the present investigation the use of organic solvent has been avoided, making the method environmentally friendly. Urea has demonstrated enhancement in aqueous solubilities of a large number of poorly water-soluble drugs, thereby widely used as a hydrotropic agent. There was more than 10-fold enhancement in the solubility of ornidazole in 10 M urea solution as compared to its solubility in distilled water. In the present investigation, hydrotropic solution of urea (10 M) was employed as solubilizing agent to solubilize the poorly water-soluble drug, ornidazole, from fine powder of its tablet dosage form for spectrophotometric determination in ultraviolet region at 319 nm. Beer's law was obeyed in the concentration range of 5-25 μg/ml in presence of urea. Presence of urea did not interfere in the analysis. Proposed method is new, rapid, simple, accurate, and reproducible. Statistical data proved the accuracy, reproducibility and the precision of the proposed method.

Compounds that cause increase in aqueous solubility are sometimes called hydrotropes. Concentrated aqueous hydrotropic solutions of urea, sodium benzoate, nicotinamide, sodium salicylate, sodium acetate and sodium citrate have been observed to enhance the aqueous solubility of many poorly water-soluble drugs[1–15]. Maheshwari *et al*.[1–10], have analyzed a large number of poorly water-soluble drugs quantitatively using hydrotropic solubilization phenomenon. Urea has demonstrated enhancement in aqueous solubilities of a large number of poorly water-soluble drugs, hence widely used as a hydrotropic agent. Ornidazole showed maximum absorbance at 319 nm and Beer's law was obeyed in the concentration range of 5-25 μg/ml in presence of urea. There was more than 10-fold enhancement in the solubility of ornidazole in 10 M urea solution as compared to its solubility in distilled water. Therefore, it was thought worthwhile to employ 10 M urea solution to solubilize the drug from fine powder of the tablets to carryout the spectrophotometric estimation at 319 nm.

Ornidazole drug sample was supplied by M/s Parenteral Drug India Limited, Indore, India. Tablets of ornidazole (Ornida, Aristo Pharmaceuticals Limited, Mandideep, India. Dazolic, Sun Pharmaceutical Industries, Dadra, India) were procured from the local Pharmacy. All other chemicals used were of analytical grade. A Shimadzu UV/Vis recording spectrophotometer (model-UV-160 A) with 1 cm matched silica cells was employed for spectrophotometric analysis.

To prepare calibration curve, about 100 mg of ornidazole bulk drug was accurately weighed and transferred to a 25 ml volumetric flask. Ten millilitres of 10 M urea solution was added and the flask was shaken to solublize the drug. Then, the volume was made up to the mark with distilled water. The stock solution was diluted with distilled water to obtain various solutions containing 5, 10, 15, 20, and 25 μg/ml drug. The absorbances of these solutions were noted at 319 nm against respective reagent blanks to obtain calibration curve.

Solubility of ornidazole in distilled water and 10 M urea were determined at 28±1°. Sufficient amount of drug was added to screw-capped 30 ml glass vials containing hydrotropic solution and distilled water. The vials were shaken mechanically for 12 h at 28±1° in an orbital flask shaker (Khera Instrument Pvt. Ltd., Delhi, India). The solutions were allowed to equilibrate for next 12 h and then centrifuged for 5 min at 2000 rpm (Remi Instruments Ltd., Mumbai, India). The supernatant of each vial was filtered through Whatman filter paper No. 41. Filtrates were diluted suitably with distilled water and analyzed spectrophotometrically at 319 nm to determine the solubilities.

For the quantitative estimation of ornidazole tablet formulations, using the proposed method of analysis, twenty tablets of formulation-1 (Ornida tablets) or formulation-2 (Dazolic tablets) were weighed and finely powdered. Powder equivalent to 100 mg of ornidazole was taken in 25 ml volumetric flask. Ten milliliters of 10 M urea solution was transferred and the flask was shaken vigorously for 10 min and made up the volume up to the mark with distilled water. After filtration through a Whatman filter paper No. 41, the filtrate was appropriately diluted with distilled water and the absorbance was noted at 319 nm against reagent blank. Using the calibration curve the drug content was calculated and reported in Table 1. The analysis was performed in triplicate.

**TABLE 1:** ANALYSIS OF COMMERCIAL TABLETS OF ORNIDAZOLE WITH STATISTICAL EVALUATION

Tablet formulation	label claim mg/tab	Per cent label claim estimated (mean±SD)	% Coefficient of variation	Standard error
1	500	98.74±0.867	0.878	0.501
2	500	99.31±1.234	1.243	0.712

* n=3, formulation 1 is Ornida tablets and formulation 2 is Dazolic tablets

To study the accuracy, reproducibility and the precision of the proposed method, recovery experiments were carried out. For this, similar procedure was followed to conduct the recovery studies taking 30 mg and 60 mg pure drug together with suitable quantities of fine powder of tablets (formulation-1 and formulation-2) equivalent to 100 mg drug and drug content was calculated. From the difference, the amount of pure drug added was obtained and the per cent recovery was calculated (Table 2). Each type of analysis was performed thrice.

**TABLE 2:** RECOVERY STUDIES WITH STATISTICAL EVALUATION

Tablet formulation	Drug present in preanalyzed tablet powder (mg)	Drug added (mg)	%Recovery estimated* (Mean±SD)	%Coefficient of variation	Standard error
1	100	30	100.72±0.881	0.875	0.509
2	100	60	99.10±1.223	1.234	0.706
1	100	30	99.18±1.543	1.556	0.891
2	100	60	98.79±0.440	0.445	0.254

* n=3, formulation 1 is Ornida tablets and formulation 2 is Dazolic tablets

In solubility determination studies, it was found that there was more than 10 fold enhancement in the solubility of ornidazole in 10 M urea solution, as compared to its solubility in distilled water at 28±1°. The observed solubilities of the drug in distilled water and 10 M urea solution were 8.03 mg/ml and 82.20 mg/ml, respectively.

As evident from Table 1, the mean per cent label claims estimated using the proposed method were 98.74 (formulation-1) and 99.31 (formulation-2), which are very close to 100, indicating the accuracy of the proposed method. The low values of statistical parameters viz. standard deviation (0.867 and 1.234), percent coefficient of variation (0.878 and 1.243) and standard error (0.501 and 0.712) validated the proposed method.

The results of recovery studies (using the proposed method) presented in Table 2 indicated that the values of mean per cent recoveries estimated in case of formulation 1 (99.10 and 100.72) and formulation 2 (98.79 and 99.18) were very close to 100. This, together with low values of standard deviation (0.440 to 1.543), percent coefficient of variation (0.445 to 1.556) and standard error (0.254 to 0.891), further proved the accuracy, reproducibility and the precision of the proposed method.

Urea is a low-cost chemical. It is harmless to use. Also, the solution of urea is non-volatile. Thus, it is concluded that the proposed method is simple, eco-friendly, cost-effective, safe, accurate and precise and can be successfully employed in the routine analysis of ornidazole tablets.

Similar to this method, other poorly water-soluble drugs can also be tried for enhancement effect on solubility in urea solution. If there is significant enhancement in solubility by hydrotropic solution of urea and the selected wavelength for spectrophotometric analysis is above 245 nm, then the hydrotropic method can be successfully employed for its estimation precluding the use of organic solvents.
